# Transmission of Middle East Respiratory Syndrome Coronavirus Infections in Healthcare Settings, Abu Dhabi

**DOI:** 10.3201/eid2204.151615

**Published:** 2016-04

**Authors:** Jennifer C. Hunter, Duc Nguyen, Bashir Aden, Zyad Al Bandar, Wafa Al Dhaheri, Kheir Abu Elkheir, Ahmed Khudair, Mariam Al Mulla, Feda El Saleh, Hala Imambaccus, Nawal Al Kaabi, Farrukh Amin Sheikh, Jurgen Sasse, Andrew Turner, Laila Abdel Wareth, Stefan Weber, Asma Al Ameri, Wesal Abu Amer, Negar N. Alami, Sudhir Bunga, Lia M. Haynes, Aron J. Hall, Alexander J. Kallen, David Kuhar, Huong Pham, Kimberly Pringle, Suxiang Tong, Brett L. Whitaker, Susan I. Gerber, Farida Ismail Al Hosani

**Affiliations:** Centers for Disease Control and Prevention, Atlanta, Georgia, USA (J.C. Hunter, D. Nguyen, N.N. Alami, S. Bunga, L.M. Haynes, A.J. Hall, A.J. Kallen, D. Kuhar, H. Pham, K. Pringle, S. Tong, B.L. Whitaker, S.I. Gerber);; Health Authority—Abu Dhabi, Abu Dhabi, United Arab Emirates (B. Aden, Z. Al Bandar, W. Al Dhaheri, K. Abu Elkheir, A. Khudair, M. Al Mulla, F. El Saleh, F.I. Al Hosany);; Abu Dhabi Health Services Company, Abu Dhabi (H. Imambaccus, N. Al Kaabi, F. Amin Sheikh, J. Sasse, A. Turner, L. Abdel Wareth, S. Weber);; Molecular Diagnostic Laboratory of Sheikh Khalifa Medical City, Abu Dhabi (A. Al Ameri, W. Abu Amer)

**Keywords:** Middle East Respiratory Syndrome coronavirus, coronavirus infections, MERS-CoV, healthcare-associated infections, viruses, United Arab Emirates, nosocomial infections, transmission, zoonoses

## Abstract

Middle East respiratory syndrome coronavirus (MERS-CoV) infections sharply increased in the Arabian Peninsula during spring 2014. In Abu Dhabi, United Arab Emirates, these infections occurred primarily among healthcare workers and patients. To identify and describe epidemiologic and clinical characteristics of persons with healthcare-associated infection, we reviewed laboratory-confirmed MERS-CoV cases reported to the Health Authority of Abu Dhabi during January 1, 2013–May 9, 2014. Of 65 case-patients identified with MERS-CoV infection, 27 (42%) had healthcare-associated cases. Epidemiologic and genetic sequencing findings suggest that 3 healthcare clusters of MERS-CoV infection occurred, including 1 that resulted in 20 infected persons in 1 hospital. MERS-CoV in healthcare settings spread predominantly before MERS-CoV infection was diagnosed, underscoring the importance of increasing awareness and infection control measures at first points of entry to healthcare facilities.

## Abstract 2

Early detection and adherence to infection prevention recommendations are necessary to avoid transmission.

Middle East respiratory syndrome coronavirus (MERS-CoV) is a novel coronavirus first identified in the Middle East region in 2012. Epidemiologic aspects of this virus remain poorly defined, but human-to-human transmission of MERS-CoV in healthcare facilities is recognized as a means of spreading infection (*1**–**7*). In Saudi Arabia, the country with the greatest number of MERS-CoV infections, exposures in healthcare facilities have resulted in repeated outbreaks and have been linked to spread of disease after the virus has been introduced from other sources (e.g., zoonotic) (*5**,**6**,**8**–**11*). The 2015 outbreak in South Korea is a dramatic example of extensive healthcare-associated transmission after a single introduction of MERS-CoV by an infected traveler; that introduction resulted in >180 documented infections in hospitals lacking adequate infection prevention measures (*12**,**13*). Because healthcare settings have the potential to contribute substantially to the spread of MERS-CoV infections, improving our understanding of infection risk and transmission patterns remains an urgent priority.

By early September 2015, a total of 74 patients with laboratory-confirmed MERS-CoV infection were reported in the United Arab Emirates (UAE); most were reported from Abu Dhabi during March–April 2014, when the Arabian Peninsula had a sharp increase in infections, primarily involving healthcare workers (HCWs) and patients with recent healthcare exposure (*8**,**9**,**11*). The extensive case investigation and contact tracing by HCWs and the Health Authority of Abu Dhabi in response to this increase provide resources to inform our understanding of MERS-CoV infections acquired and spread in healthcare settings. We describe the epidemiologic and clinical characteristics of healthcare-associated MERS-CoV infections in Abu Dhabi and characterize the size and suspected transmission patterns in healthcare settings.

## Methods

### Setting

Abu Dhabi is the largest of the 7 emirates of UAE. It has ≈2.3 million residents, including 1.9 million expatriates, and 35 hospitals (*14*).

### Case and Contact Investigation Methods

In January 2013, a standardized public health protocol for MERS-CoV response was established in Abu Dhabi. Case-patients were defined as persons reported to the Health Authority of Abu Dhabi with laboratory confirmation of MERS-CoV infection by PCR performed on a respiratory sample (*15**,**16*). Our analysis included all case-patients reported during January 1, 2013–May 9, 2014. Activities involved in this investigation were reviewed by the US Centers for Disease Control and Prevention (CDC) and by the Health Authority of Abu Dhabi and were determined to be an urgent public health response that did not constitute human subjects research.

Health Authority staff conducted detailed investigations of case-patients and their close contacts, interviewing case-patients or family proxies to collect demographic, clinical, and risk-factor information during the 14 days before illness onset. Additional information about clinical exposures was collected from HCW case-patients (e.g., use of personal protective equipment [PPE]). After laboratory confirmation, all case-patients were hospitalized with airborne precautions until they had 2 consecutive PCR-negative MERS-CoV tests on specimens collected at least 48 hours apart. Close contacts, which included anyone who lived with, visited, provided care for, or had other similarly close contact with case-patients while they were symptomatic, were identified from interviews and other sources (e.g., hospital documentation). The contacts were interviewed and had nasopharyngeal, sputum, or tracheal aspirate samples collected for PCR testing, regardless of symptoms. 

### Sources of Exposure

Medical and public health records were used to categorize sources of exposure. Infections of case-patients who worked at, were admitted to, or visited a healthcare facility during the 14 days before symptom onset were considered to be healthcare-associated if exposure to a known MERS-CoV case-patient occurred exclusively in this setting. Healthcare exposure to a case-patient was characterized as either confirmed (i.e., persons who had been within 2 m of a symptomatic case) (*15**,**17*) or probable (i.e., persons who had been in the same hospital unit for >1 hour, had a common HCW, or had moved into a bed or dialysis station vacated by a symptomatic case) (*18*). Probable exposure was assumed for continuously hospitalized case-patients in whom symptoms of infection developed >14 days after admission.

### Identification and Description of Clusters

Case investigations were used to construct cluster diagrams depicting suspected healthcare-associated transmission pathways. Healthcare-associated clusters were defined as >1 epidemiologically related, healthcare-associated case-patient in the same healthcare setting (i.e., with confirmed or probable exposure). Healthcare-associated clusters consisted of >1 source case (i.e., case-patient with the earliest date of symptom onset in the healthcare-associated cluster) and >1 secondary case (i.e., case-patient with confirmed exposure to the source case). Healthcare-associated clusters could also include tertiary and quaternary cases (i.e., case-patients exposed only to secondary and tertiary cases, respectively). Clinical records were used to assess probable exposures for healthcare-associated case-patients with no confirmed exposure; cases with confirmed exposure were not assessed for probable exposure because confirmed exposure was assumed to confer the greatest risk.

### Laboratory Analysis

#### PCR

Nasopharyngeal swab, sputum, or tracheal aspirate samples were tested at a central laboratory (Molecular Diagnostic Laboratory at Sheikh Khalifa Medical City Hospital) in Abu Dhabi by using real-time reverse transcription PCR (rRT-PCR) for the upstream E gene and open reading frame 1 (*19**,**20*). A convenience sample of positive isolates was validated by using the nucleocapsid-based rRT-PCR assay at CDC (*21*).

#### Sequencing

Genetic sequencing was performed on a subset of isolates from 8 case-patients (7 from healthcare-associated clusters and 1 non–healthcare related). Full genome sequencing from original respiratory samples was determined by using the Sanger method (direct genome walking PCR) and next-generation sequencing approaches (Illumina MiSeq sequencer, http://www.illumina.com/systems/miseq.html) (*22**,**23*). Sequences were aligned by using MUSCLE (*24*) within the MEGA5 program (*25*).

### Statistical Analysis

Descriptive analysis of healthcare-associated cases and clusters was conducted by using SAS version 9.3 (SAS Institute, Inc., Cary, NC, USA). Fisher exact test and independent *t*-test were used to compare clinical and demographic characteristics of source-cases and healthcare-associated cases; a 2-sided α level of 0.05 was used to determine significance.

## Results

### Case and Contact Investigation

Of 65 MERS-CoV case-patients identified during our investigation period (July 1, 2013–May 9, 2014) in Abu-Dhabi, 27 (42%) were healthcare associated; 19 (70%) of the 27 were HCWs; 6 (22%) were hospitalized patients, and 2 (7%) were hospital visitors (Table 1). An additional 16 case-patients had worked at or visited a healthcare facility in the month before illness but did not meet the healthcare-associated case definition and were excluded from this analysis; 8 of the 16 excluded case-patients were HCWs with confirmed exposure to a symptomatic case-patient outside the healthcare setting (i.e., household); 8 had visited a healthcare facility but had no probable or confirmed exposure in this setting. 

**Table 1:** Descriptive epidemiology of 30 cases of MERS-CoV infection transmitted in healthcare settings, Abu Dhabi, January 1, 2013–May 9, 2014*

Demographic and clinical characteristic	Source case-patients, n = 3‡	Healthcare-associated case-patients†				Signif§
		All HCA case-patients, n = 27	HCWs, n = 19	Patients, n = 6	Visitors, n = 2	
Median age, y (range)	59 (30–83)	43 (27–82)	39 (27–63)	65 (40–73)	44 (34–54)	
Male sex	3 (100)	17 (63)	11 (58)	5 (83)	1 (50)	
Expatriate¶	1 (33)	26 (96)	18 (95)	6 (100)	2 (100)	0.02
Exposures within 14 d before symptom onset#						
Travel	0	2 (7)	1 (5)	0	1 (50)	
Camel	2 (67)	0	0	0	0	0.01
Symptoms						
Any symptoms reported	3 (100)	16 (59)	10 (53)	5 (83)	1 (50)	
Documented fever or symptom of respiratory illness**	3 (100)	13 (48)	8 (42)	5 (83)	0	
Documented fever (≥38.5°C)	3 (100)	9 (33)	6 (32)	3 (50)	0	
Shortness of breath	3 (100)	5 (19)	0	5 (83)	0	0.01
Fatigue/malaise	2 (67)	8 (30)	4 (21)	3 (50)	1 (50)	
Cough	2 (67)	7 (26)	4 (21)	3 (50)	0	
Cough with sputum production	2 (67)	2 (7)	0	2 (33)	0	0.04
Rhinorrhea	2 (67)	2 (7)	2 (11)	0	0	0.04
Muscle aches	2 (67	7 (26	5 (26)	1 (17)	1 (50)	
Chest pain	1 (33)	2 (7)	1 (5)	1 (17)	0	
Joint pain	2 (67)	2 (7)	2 (11)	0	0	0.04
Headache	2 (67)	4 (15)	3 (16)	1 (17)	0	
Sore throat	1 (33)	5 (19)	5 (26)	0	0	
Wheezing	1 (33)	3 (11)	1 (5)	2 (33)	0	
Vomiting/nausea	1 (33	1 (4)	0	1 (17)	0	
Medical history						
Any underlying conditions	2 (67)	15 (56)	7 (37)	6 (100)	2 (100)	
Diabetes mellitus	1 (33)	6 (22)	1 (5)	4 (67)	1 (50)	
Dementia	1 (33)	0	0	0	0	
Malignancy	1 (33)	0	0	0	0	
Receiving immunosuppressant	1 (33)	0	0	0	0	
Chronic pulmonary disease	0	2 (7)	0	2 (33)	0	
Renal disease	0	5 (19)	0	4 (67)	1 (50)	
Congestive heart failure	0	1 (4)	0	1 (17)	0	
Obese††	0	2 (7)	1 (5)	1 (17)	0	
Hypertension	0	12 (44)	5 (26)	5 (83)	2 (100)	
Hyperlipidemia	0	7 (26)	4 (21)	2 (33)	1 (50)	
Asthma	0	2 (7)	2 (11)	0	0	
Ischemic heart disease	0	3 (11)	1 (5)	2 (33)	0	
Severity of symptoms						
Care in ICU	3 (100)	5 (19)	0	5 (83)	0	0.01
Supplemental O_2_ required	3 (100)	6 (22)	0	6 (100)	0	0.02
Intubated	3 (100)	3 (11)	0	3 (50)	0	<0.01
Died	2 (67)	2 (7)	0	2 (33)	0	0.04
Reason tested for MERS-CoV						
Screening as part of contact investigation	0	24 (89)	19 (100)	3 (50)	2 (100)	<0.01
Symptoms consistent with MERS-CoV	3 (100)	3 (11)	0	3 (50)	0	<0.01

*Values are no. (%) patients except as indicated. HCA, healthcare-associated; HCWs, healthcare workers; MERS-CoV, Middle East respiratory syndrome coronavirus; Signif, statistically significant.  †HCA case-patients include HCWs, patients, and hospital visitors but does not include source case-patients.  ‡Source case-patients are those with the earliest date of onset of symptoms in an HCA cluster of case-patients. §Comparison between type of case (source case vs. HCA case) determined by Fisher exact test. Only significant values are shown. ¶Nationalities: Philippines, India, Somalia, Bangladesh, Egypt, Jordan, Oman, Pakistan, Sudan, and Syria. #For case-patients with no reported symptoms, date of positive sample collection was used in place of symptom onset. **Symptoms of respiratory illness are cough, shortness of breath, or wheezing.  ††Obesity status was determined by clinical staff.

### Source Cases

All 3 source case-patients in the healthcare-associated clusters were men with a median age of 59 years; 2 had a history of camel exposure in the 14 days before symptom onset (Table 1). All were symptomatic, were admitted to intensive care, required supplemental oxygen, and were intubated; 2 died (67% case-fatality rate).

### Healthcare-Associated Cases

Of the 27 healthcare-associated case-patients, 17 (63%) were male; median age was 43 years. None had a history of camel exposure during the 14 days before symptom onset. Disease severity varied by type of case; source case-patients had the greatest disease severity (Table 1). Fewer than half (42%) of HCW case-patients reported fever or symptoms of respiratory disease, and none required intensive care. The proportion of patients who died was significantly lower among healthcare-associated case-patients (2/27 [7%]) than among source case-patients (2/3 [67%]); death among healthcare-associated case-patients occurred only among hospital patients (2/6 [33%]).

### Identification and Description of Clusters

**Figure 1:**
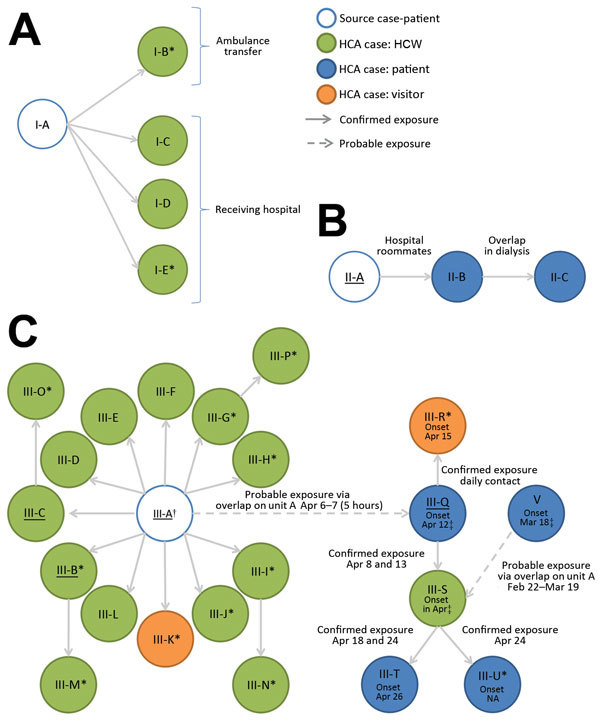
Transmission of Middle East respiratory syndrome coronavirus (MERS-CoV) infections in 3 healthcare setting clusters, Abu Dhabi, January 2013–May 2014. A) Cluster I; B) cluster II; C) cluster III. Individual patients are identified by cluster and a letter indicating the order in which cases occurred (e.g., I-A indicates the source case-patient for cluster I). Figure panels illustrate suspected chains of transmission of MERS-CoV infection within the 3 clusters. Each circle represents a case-patient. Arrows connect case-patients with likely source of MERS-CoV infection, with arrows pointing in the direction of transmission (i.e., from source case-patient to secondary case-patient). Descriptions adjacent to arrows indicate the timing or location of confirmed (shown with solid arrows) and probable (shown with broken arrows) exposures between the case-patients. Asterisks (*) indicate case-patients who reported no fever or symptoms of respiratory disease; underlining indicates cases for which isolates underwent genetic sequencing. †Dates of exposure and symptom onset for case-patients III-B–III-L are summarized in Figure 2. ‡After identification of MERS-CoV in case-patient V, healthcare workers in unit A were screened beginning March 24, 2014. MERS-CoV was not detected from a sputum specimen collected from case-patient III-S at this time. The MERS-CoV–positive specimen was collected on April 24, after identification of case-patient III-Q on the same ward. HCA, healthcare-associated; HCW, healthcare worker.

From the epidemiologic and genetic investigation, we identified 3 healthcare-associated clusters at 3 hospitals during our investigation period. The clusters ranged in size from 3 to 21 case-patients (Figure 1).

#### Cluster I, July 2013

The source case-patient for cluster I (patient I-A; Figure 1) was an 82-year-old UAE resident who owned a farm with camels, had no travel history or contact with another case-patient, and was hospitalized in Abu Dhabi with respiratory symptoms in July 2013. Two days later, the patient was transferred by ambulance to another hospital ≈350 km away, where he tested positive by PCR for MERS-CoV, developed acute respiratory distress syndrome, and died.

Among 277 healthcare contacts identified in the 2 hospitals and among transport staff, 4 healthcare-associated case-patients were detected through PCR screening of respiratory specimens, including the nurse who accompanied the source case-patient in the ambulance (patient I-B; Figure 1) and 3 HCWs who were involved in the patient’s evaluation or early care at the second hospital (1 physician, 2 nurses; patients I-C, I-D, and I-E; Figure 1). All infected HCWs had close contact with the case-patient without respiratory protection before the MERS-CoV diagnosis.

#### Cluster II, March–April 2014

The source case-patient for cluster II (patient II-A; Figure 1) was a 68-year-old UAE resident who owned a farm and reported direct contact with camels. He had no travel history, no contact with a known case, and no healthcare facility contact during the 14 days before symptom onset. In March 2014, this patient was hospitalized in Abu Dhabi with respiratory symptoms; MERS-CoV was diagnosed 4 days later.

Among 90 healthcare contacts identified, 2 healthcare-associated case-patients were detected. A secondary case-patient (patient II-B; Figure 1) who shared a room with the symptomatic source case-patient before the MERS-CoV diagnosis subsequently developed respiratory symptoms, was readmitted to the hospital, was diagnosed with MERS-CoV, and died. Screening of contacts identified a tertiary case-patient (patient II-C; Figure 1) who had a probable exposure to patient II-B in hemodialysis (before diagnosis) and no exposure to the source case-patient.

#### Cluster III, March–April 2014

The source case-patient for cluster III (patient III-A; Figure 1) was a 45-year-old expatriate who had no travel history, no animal contact, and no healthcare facility contact during the 14 days before symptom onset. He worked in the storage room at a paramedic dispatch station, a nonclinical facility located in a police station where no patient contact occurs. An extensive epidemiologic investigation of household and work contacts revealed no known exposure to a case before symptom onset and no link to cluster II, which occurred at a hospital >350 km away. No known case-patients were transported by paramedics in this unit.

On March 29, the source case-patient developed respiratory symptoms. From April 2–6, he was assessed at an emergency department (ED) in Abu Dhabi on 3 occasions for fever, cough, shortness of breath, and pneumonia. He was examined in an ED room (with a curtain divider) under standard precautions and was given a surgical mask and oxygen, which staff reported he removed repeatedly because of difficulty breathing. On April 6, he was admitted to a general medical unit (unit A), where he received care for 5 hours before being transferred to the intensive care unit (ICU) and placed on airborne infection isolation precautions. A MERS-CoV diagnosis was confirmed on April 9, and he died the next day.

**Figure 2:**
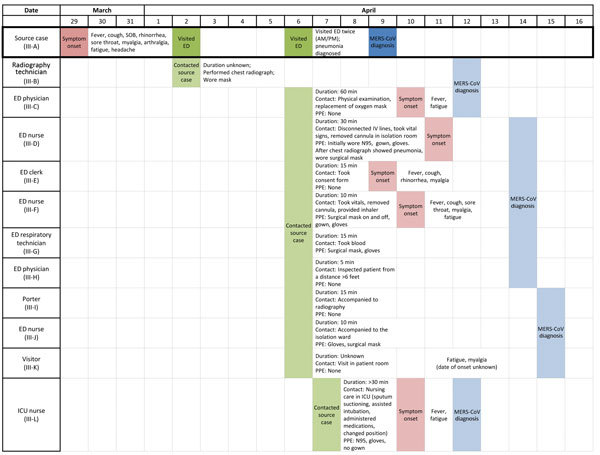
Timeline of exposures, symptom onset, and diagnosis of Middle East respiratory syndrome coronavirus (MERS-CoV) among secondary case-patients in a healthcare-associated cluster (cluster III), Abu Dhabi, 2014. Colored boxes indicate key dates for each case-patient: green boxes indicate date of interaction between source case (patient III-A) and healthcare providers; pink boxes indicate date of symptom onset; blue boxes indicate date of MERS-CoV diagnosis. For 5 case-patients who reported no symptoms, symptom onset is not listed; data exclude a secondary case with probable exposure (patient III-Q). SOB, shortness of breath; ICU, intensive care unit; PPE, personal protective equipment; duration, duration of exposure; ED, emergency department.

PCR screening of respiratory specimens from 224 possible healthcare contacts from the ED, ICU, and medical wards identified 15 healthcare-associated case-patients (Figure 1). Ten were secondary case-patients who had exposure to the source case-patient during ED visits (patient III-B on April 2; patients III-C–K on April 6); 1 was a HCW who cared for the source case-patient in the ICU after the MERS-CoV diagnosis (patient III-L); and 4 were tertiary case-patients among HCWs who had no exposure to the source case-patient but had confirmed exposure to infected co-workers in radiology (patient III-M), the hospital transport unit (patient III-N), and the ED (patients III-O and III-P) (Figure 2). Attack rates among healthcare contacts with confirmed exposure to the source case-patient were estimated to be 16% (10/64 contacts) in the ED and radiology department before diagnosis and 5% (1/21 contacts) in the ICU after diagnosis.

A second subcluster of illnesses was detected in unit A (i.e., general medical unit of hospital of admission) when a 74-year-old hospital patient (patient III-Q) who had been admitted to the unit in February developed new onset of shortness of breath on April 12 and had a MERS-CoV–positive sample collected the same day. During the 14 days before symptom onset, the patient was continuously hospitalized, had no travel history, no animal contact, and no confirmed exposure to a case-patient; however, she had resided in a room adjacent to patient III-A (source case-patient of cluster III) in unit A for 5 hours on April 6–7. During this period, no close contact with patient III-A occurred, and no documented common healthcare contacts or common equipment was identified; consequently, this case constitutes a probable rather than confirmed exposure.

Subsequent screening of 83 healthcare contacts of patient III-Q identified 2 healthcare-associated case-patients, including patient III-Q’s daughter (patient III-R, tertiary case), who had been staying in the patient’s hospital room, and a nurse who provided care to patient III-Q in unit A (patient III-S, tertiary case). Further screening of 12 patients who received care from the infected nurse while she was symptomatic identified 2 more case-patients (patients III-T and III-U, quaternary cases), who were bedbound chronic care patients hospitalized for >4 weeks before their MERS-CoV–positive sample collection date. No cases were identified among the 50 additional healthcare contacts screened from Unit A; these contacts included the remaining 42 HCWs who worked on Unit A and the 8 patients who had shared a room with patient III-T. In total, 20 healthcare-associated cases (12 secondary, 6 tertiary, and 2 quaternary) are attributable to a single introduction in the hospital.

A final healthcare-associated case-patient who was also cared for in unit A was identified (patient V; Figure 1). This 40-year-old expatriate man was admitted to the hospital in February 2014 with shortness of breath and multiple concurrent conditions, including congestive heart failure. The patient was cared for in unit A and a dialysis unit during February–March; new fever and shortness of breath developed on March 18, and he tested positive for MERS-CoV and was transferred to the ICU, where he died. The case-patient was hospitalized during the 14 days before symptom onset and had no travel history, animal contact, or contact with a known case. No source case or secondary cases were identified when 141 healthcare-associated contacts were screened (including patient III-S, a HCW who submitted a PCR-negative respiratory specimen during this contact investigation).

### Laboratory

#### PCR

All case isolates were laboratory confirmed as positive by rRT-PCR for the upstream E gene and open reading frame 1. Average time between sample collection and laboratory results was 1 day (range 0–3 days). All 23 PCR-confirmed case isolates included in the validation sample were verified by using the N2 assay at CDC.

#### Sequencing

Genetic sequencing was performed on a subset of 8 isolates: 7 from case-patients in healthcare-associated clusters (patients II-A, III-A–III-C, III-O, III-Q, and III-R) and 1 from a non–healthcare-related case-patient from Abu Dhabi (Table 2). Full genome sequences were deposited in GenBank (accession nos. KP209306–KP209313). The genome sequences are similar (>99%) to other known MERS-CoV and clusters most closely with camel-derived MERS-CoV strains (GenBank accession nos. KJ650295–KJ650297) obtained in Al-Hasa, Saudi Arabia, in 2013, suggesting potential camel origin. Comparing complete genome sequences to the source case for cluster III (patient III-A) showed that all 6 MERS-CoV sequences from cluster III are considered to be genetically related, with <2 nt differences in the genome. The sequence for the source case from cluster II (patient II-A) is not considered related (11-nt difference). The non–healthcare-associated case from 2013 is even more divergent (30-nt difference).

**Table 2:** Nucleotide sequence variations of MERS-CoV full genomes from 8 case-patients in Abu Dhabi, January 1, 2013–May 9, 2014*

**Genome position, nt**	**Patients associated with healthcare clusters**							**Patient V†**
	III-A	III-B	III-C	III-O	III-Q	III-R	II-A	
**381**	C							T
**1,226**	T						C	C
**2,015**	T						C	
**3,110**	C							T
**3,280**	T						C	C
**3,799**	G							A
**3,968**	C							T
**4,625**	C							T
**5,065**	T							C
**5,152**	A			G				
**5,381**	C							T
**6,189**	C							T
**7,124**	G						T	T
**7,610**	C						T	
**11,631**	C							T
**11,766**	T							C
**11,785**	Y (T/C)	C	T	T	T	T	C	C
**13,331**	T						C	
**15,592**	A							G
**16,381**	A	C						
**18,045**	T							C
**18,208**	T							C
**18,966**	T	G						
**19,072**	C						T	
**21,382**	T		C					
**21,531**	T							G
**21,777**	G		A					
**22,394**	C							T
**22,760**	C	Y (T/C)						
**22,790**	T							C
**22,913**	T						C	
**23,549**	G							A
**23,685**	A							C
**23,883**	G							A
**24,518**	G							A
**24,602**	C							T
**24,687**	T							C
**25,364**	C							T
**26,672**	G						T	
**27,204**	T							A
**27,206**	C							A
**27,208**	A							T
**27,211**	C							A
**27,867**	G						T	
**29,170**	G						T	
**Total nt differences**		2	2	1	0	0	11	30

*Genome sequences compared with those for case III-A, the source case-patient for healthcare-associated cluster III. The variation table was generated on the basis of the full genome sequence described in the Methods section. Blank cells indicate no sequence difference. MERS-CoV, Middle East respiratory syndrome coronavirus. **†Case not associated with healthcare.**

### Infection Prevention

Of the 14 HCWs (patients I-B–I-E, III-B–III-J, and III-L) who became infected with MERS-CoV after caring for a source case-patient, 13 (93%) were exposed before the patient’s diagnosis. PPE use during care was inconsistent among these HCWs (Table 3). The 1 HCW who became infected after caring for a recognized case-patient reported use of gloves and N95 respirator masks during all patient care activities but did not consistently wear a gown and recalled an occasion when patient material contaminated her clothing (Table 3).

**Table 3:** Healthcare interactions for 14 healthcare workers who became infected with MERS-CoV after caring for a source case-patient, Abu Dhabi, January 1, 2013–May 9, 2014*

**Description of healthcare interaction**	**Healthcare workers, no. (%)**
**Timing of interaction**	
** Before MERS-CoV diagnosis in source case-patient**	13 (93)
**Type of interaction†**	
** Patient examination**	7 (50)
** Procedure with potential aerosol generation‡**	5 (36)
** Patient transport**	3 (21)
** Radiograph**	1 (7)
** Clerical**	1 (7)
** Unknown**	1 (7)
**Duration of interaction†**	
** <10 min**	1 (11)
** 10–30 min**	6 (43)
** >30 min**	2 (22)
** Unknown**	5 (36)
**PPE use during interaction†§**	
** Any mask**	6 (43)
** Surgical mask**	5 (36)
N95 respirator**¶**	2 (14)
** Gloves**	4 (29)
** Gown**	3 (21)
** Gown, gloves, and surgical mask or N95 respirator**	3 (21)

*Of the 19 healthcare worker case-patients identified, 14 occurred in persons who provided care for a source case (cases I-B–I-E, III-B–III-J, III-L); these 14 healthcare workers are described here. MERS-CoV, Middle East respiratory syndrome coronavirus; PPE, personal protective equipment.  †Self-reported information on eye protection is not available. ‡Manipulation of cannula or oxygen mask (n = 3), administration of inhaler or nebulizer treatment (n = 2), intubation (n = 1), suctioning before intubation (n = 1); healthcare workers could perform >1 of these patient care activities. §Information on eye protection is not available. **¶**Of the 2 healthcare workers who reported wearing an N95 respirator, 1 wore N95 inconsistently, and 1 reported wearing gloves and a respirator during all patient care activities but did not consistently wear a gown and recalls an occasion when patient material contaminated her clothing.

## Discussion

MERS-CoV in healthcare settings accounts for >40% of all reported infections in Abu Dhabi. We found that healthcare-associated transmission occurred predominantly when HCWs, patients, and visitors were exposed to an infected person before recognition of MERS-CoV and implementation of appropriate infection prevention measures. These findings underscore the importance of early detection and intervention to limit spread of disease.

In the largest healthcare cluster in our investigation, 1 patient appears to have directly infected 12 persons in 1 hospital, resulting in a total of 20 healthcare-associated infections caused by secondary, tertiary, and quaternary transmission. Among ED HCWs, we estimate a 16% attack rate, ≈4 times higher than average household transmission estimates (*4*). Our findings add to previously reported examples of more extensive transmission occurring in healthcare facilities in South Korea, Saudi Arabia, and Jordan (*5**,**6**,**11**–**13*) and suggest that, in the absence of appropriate infection prevention measures, healthcare settings may be particularly efficient for MERS-CoV transmission. As described during an outbreak of severe acute respiratory syndrome, transmission in healthcare settings may be increased by various factors: higher than usual infectiousness of patients because of high viral loads or presence of symptoms that increase shedding; use of procedures that aerosolize infectious respiratory illness; close patient–HCW proximity during medical encounters; and other not-yet-identified factors (*26*). Extensive contact-tracing practices in Abu Dhabi, including testing contacts of case-patients regardless of symptoms, and whole-genome sequencing were essential for fully characterizing the transmission patterns in this cluster.

In our investigation, nearly all healthcare-associated transmission events occurred before MERS-CoV was suspected and diagnosed. After diagnosis, >500 patient-days of hospital care were provided to case-patients in Abu Dhabi; among HCWs providing this care, 1 infection occurred in an ICU nurse who reported not fully adhering to recommended prevention measures while she cared for a case-patient. Although delayed diagnosis contributed to all other transmission events, reasons for delays varied, highlighting challenges faced by the healthcare community: 1 patient was infected early in the outbreak, before high visibility of MERS-CoV and prevention policies; 1 patient sought care from an ED but had no known epidemiologic risk factors for MERS-CoV; 1 hospitalized patient had MERS-CoV symptoms that could be explained by other concurrent conditions; 1 infected HCW with mild illness did not report symptoms and continued working while ill. In the largest cluster, despite strong hospital and public health policies for triaging and isolating patients with respiratory symptoms as part of the MERS-CoV response, the source case-patient was placed under standard precautions, rather than contact and airborne precautions (*27*). Reasons for not implementing protocols in this instance are unknown, but the patient’s lack of known risk factors (e.g., exposure to a case-patient) likely contributed to low clinical suspicion. This cluster underscores the importance of maintaining vigilance and adherence to infection prevention policy, particularly in regions where known MERS-CoV infections exist.

The severity of illness associated with MERS-CoV infection among case-patients in our investigation ranged from asymptomatic to severe disease, as has been previously reported (*4**,**5**,**10**,**18*). Severity of symptoms varied by type of case; death occurred among 2 of 3 source case-patients, 1 of 3 infected hospital patients, and no infected HCWs, who typically reported mild or no symptoms. We identified 3 instances in which transmission appears to have occurred from infected HCWs who reported no fever or symptoms of respiratory illness (patients III-B, III-G, and III-I). Although underreporting of symptoms or failure to recognize exposures cannot be ruled out, our findings show that increased understanding of transmission risks for persons with mild disease and improved strategies for early detection of illness are needed (*1**,**4**,**7*).

This investigation has several limitations. Although the healthcare clusters we describe are supported epidemiologically and steps were taken to ensure that clusters were conservatively constructed (e.g., excluding healthcare workers with known exposures in the community), other transmission pathways cannot be excluded. Genetic sequencing of a limited number of cases supports the epidemiologic characterization of cases and clusters, but we were unable to sequence and assess the molecular relatedness of all case isolates, a step that previous investigations have shown to be informative (*11**,**28*). On the basis of the current understanding of the mutation rate of MERS-CoV, the genetic differences observed in case isolates from clusters II and III are consistent with 2 separate introductions (*11*); however, we cannot rule out the possibility that these clusters are related. Furthermore, transmission pathways were generated on the basis of self-reporting or other documentation, and exposures may have been missed or forgotten. Such lapses might explain the difficulty in ascertaining a source of exposure for 1 healthcare-associated case (patient V). In addition, follow-up serologic testing for MERS-CoV–specific antibodies and repeat PCR testing were not performed on healthcare-associated contacts, so additional cases may have been missed. Last, by restricting our definition of healthcare-associated cases to persons with recognized exposures in healthcare settings, we may underestimate the true number of cases, particularly if sources of infection (i.e., case-patients) went undetected. Because our objective was to characterize transmission patterns among known healthcare-associated cases, we considered the conservative definition to be most appropriate.

In conclusion, large healthcare clusters of MERS-CoV illness contribute to substantial illness and also have potential for secondary consequences, including fear among HCWs and the public. MERS-CoV can clinically appear with mild or nonspecific respiratory symptoms, and patients may seek care without having known risk factors for infection. Maintaining a high index of suspicion in every patient encounter, especially at first points of patient entry such as EDs or primary healthcare settings, is imperative, particularly in regions reporting MERS-CoV cases. Early detection of cases, full adherence to infection prevention recommendations, and recognition of illness among HCWs are necessary factors to prevent further transmission of MERS-CoV in healthcare settings. Supporting healthcare facilities in these efforts remains a priority.

## References

[1] Memish ZA, Zumla AI, Assiri A. Middle East respiratory syndrome coronavirus infections in health care workers. N Engl J Med. 2013;369:884–6. 10.1056/NEJMc1308698 23923992

[2] Memish ZA, Zumla AI, Al-Hakeem RF, Al-Rabeeah AA, Stephens GM. Family cluster of Middle East respiratory syndrome coronavirus infections. N Engl J Med. 2013;368:2487–94. 10.1056/NEJMoa1303729 23718156

[3] Zaki AM, van Boheemen S, Bestebroer TM, Osterhaus AD, Fouchier RA. Isolation of a novel coronavirus from a man with pneumonia in Saudi Arabia. N Engl J Med. 2012;367:1814–20. 10.1056/NEJMoa1211721 23075143

[4] Drosten C, Meyer B, Muller MA, Corman VM, Al-Masri M, Hossain R, Transmission of MERS-coronavirus in household contacts. N Engl J Med. 2014;371:828–35. 10.1056/NEJMoa1405858 25162889

[5] Al-Abdallat MM, Payne DC, Alqasrawi S, Rha B, Tohme RA, Abedi GR, Hospital-associated outbreak of Middle East respiratory syndrome coronavirus: a serologic, epidemiologic, and clinical description. Clin Infect Dis. 2014;59:1225–33. 10.1093/cid/ciu359 24829216 PMC4834865

[6] Assiri A, McGeer A, Perl TM, Price CS, Al Rabeeah AA, Cummings DA, Hospital outbreak of Middle East respiratory syndrome coronavirus. N Engl J Med. 2013;369:407–16. 10.1056/NEJMoa1306742 23782161 PMC4029105

[7] Omrani AS, Matin MA, Haddad Q, Al-Nakhli D, Memish ZA, Albarrak AM. A family cluster of Middle East respiratory syndrome coronavirus infections related to a likely unrecognized asymptomatic or mild case. Int J Infect Dis. 2013;17:e668–72. 10.1016/j.ijid.2013.07.001 23916548 PMC7110537

[8] Oboho IK, Tomczyk SM, Al-Asmari AM, Banjar AA, Al-Mugti H, Aloraini MS, 2014 MERS-CoV outbreak in Jeddah—a link to health care facilities. N Engl J Med. 2015;372:846–54. 10.1056/NEJMoa1408636 25714162 PMC5710730

[9] Drosten C, Muth D, Corman VM, Hussain R, Al Masri M, Hajomar W, et al. An observational, laboratory-based study of outbreaks of Middle East respiratory syndrome coronavirus in Jeddah and Riyadh, kingdom of Saudi Arabia, 2014. Clin Infect Dis. 2015;60:369–77. 10.1093/cid/ciu812 25323704 PMC4303774

[10] Assiri A, Al-Tawfiq JA, Al-Rabeeah AA, Al-Rabiah FA, Al-Hajjar S, Al-Barrak A, Epidemiological, demographic, and clinical characteristics of 47 cases of Middle East respiratory syndrome coronavirus disease from Saudi Arabia: a descriptive study. Lancet Infect Dis. 2013;13:752–61. 10.1016/S1473-3099(13)70204-4 23891402 PMC7185445

[11] Fagbo SF, Skakni L, Chu DK, Garbati MA, Joseph M, Peiris M, Molecular epidemiology of hospital outbreak of Middle East respiratory syndrome, Riyadh, Saudi Arabia, 2014. Emerg Infect Dis. 2015;21:1981–8. 10.3201/eid2111.150944 26484549 PMC4622263

[12] World Health Organization. Summary and risk assessment of current situation in Republic of Korea and China. 2015 Jun 19 [cited 2015 Jun 24]. http://www.who.int/csr/disease/coronavirus_infections/risk-assessment-19june2015/en/

[13] Cowling BJ, Park M, Fang VJ, Wu P, Leung GM, Wu JT. Preliminary epidemiological assessment of MERS-CoV outbreak in South Korea, May to June 2015. Euro Surveill. 2015;20:pii: 21163. 10.2807/1560-7917.ES2015.20.25.21163 26132767 PMC4535930

[14] Statistics Centre Abu Dhabi. Statistical yearbook of Abu Dhabi 2013 [cited 2014 Oct 1]. https://www.scad.ae/en/Pages/ThemeReleaseDetail.aspx?ReleaseID=213&ThemeID=1

[15] Centers for Disease Control and Prevention. Middle East respiratory syndrome (MERS). Interim patient under investigation (PUI) guidance and case definitions. 2015 Dec 8 [cited 2015 Dec 8]. http://www.cdc.gov/coronavirus/mers/case-def.html

[16] Health Authority–Abu Dhabi (HAAD). Circular DG 15/14. Novel Middle East respiratory syndrome coronavirus (MERS-CoV) clinical care pathway (update). 2014 Apr 23 [cited 2014 Oct 1]. http://www.haad.ae/HAAD/LinkClick.aspx?fileticket=TQ1IK3uyMC4%3D&tabid=207

[17] World Health Organization (WHO). Assessment of potential risk factors of infection of Middle East respiratory syndrome coronavirus (MERS-CoV) among health care personnel in a health care setting. Version 1. 2014 Jan 27 [cited 2014 Oct 1]. http://www.who.int/csr/disease/coronavirus_infections/Healthcare_MERS_Seroepi_Investigation_27Jan2014.pdf?ua=1

[18] Assiri A, McGeer A, Perl TM, Price CS, Al Rabeeah AA, Cummings DA, Hospital outbreak of Middle East respiratory syndrome coronavirus. N Engl J Med. 2013;369:407–16. 10.1056/NEJMoa1306742 23782161 PMC4029105

[19] Corman VM, Eckerle I, Bleicker T, Zaki A, Landt O, Eschbach-Bludau M, Detection of a novel human coronavirus by real-time reverse-transcription polymerase chain reaction. Euro Surveill. 2012;17:pii: 20285. 23041020 10.2807/ese.17.39.20285-en

[20] Corman VM, Muller MA, Costabel U, Timm J, Binger T, Meyer B, Assays for laboratory confirmation of novel human coronavirus (hCoV-EMC) infections. Euro Surveill. 2012;17:pii: 20334. 23231891 10.2807/ese.17.49.20334-en

[21] Lu X, Whitaker B, Sakthivel SK, Kamili S, Rose LE, Lowe L, Real-time reverse transcription-PCR assay panel for Middle East respiratory syndrome coronavirus. J Clin Microbiol. 2014;52:67–75. 10.1128/JCM.02533-13 24153118 PMC3911421

[22] Chu DK, Poon LL, Gomaa MM, Shehata MM, Perera RA, Abu Zeid D, MERS coronaviruses in dromedary camels, Egypt. Emerg Infect Dis. 2014;20:1049–53. 10.3201/eid2006.140299 24856660 PMC4036765

[23] Cotten M, Lam TT, Watson SJ, Palser AL, Petrova V, Grant P, Full-genome deep sequencing and phylogenetic analysis of novel human betacoronavirus. Emerg Infect Dis. 2013;19:736–42. 10.3201/eid1905.130057 23693015 PMC3647518

[24] Edgar RC. MUSCLE: multiple sequence alignment with high accuracy and high throughput. Nucleic Acids Res. 2004;32:1792–7. 10.1093/nar/gkh340 15034147 PMC390337

[25] Tamura K, Peterson D, Peterson N, Stecher G, Nei M, Kumar S. MEGA5: molecular evolutionary genetics analysis using maximum likelihood, evolutionary distance, and maximum parsimony methods. Mol Biol Evol. 2011;28:2731–9. 10.1093/molbev/msr121 21546353 PMC3203626

[26] McDonald LC, Simor AE, Su I-J, Maloney S, Ofner M, Chen K-T, SARS in healthcare facilities, Toronto and Taiwan. Emerg Infect Dis. 2004;10:777–81. 10.3201/eid1005.030791 15200808 PMC3323242

[27] Health Authority–Abu Dhabi. HAAD standard for prevention and control of influenza and influenza-like illness. 2014 Mar 9 [cited 2014 Oct 1]. http://www.haad.ae/HAAD/LinkClick.aspx?fileticket=ilWNev9u9ps%3d&tabid=819

[28] Cotten M, Watson SJ, Kellam P, Al-Rabeeah AA, Makhdoom HQ, Assiri A, Transmission and evolution of the Middle East respiratory syndrome coronavirus in Saudi Arabia: a descriptive genomic study. Lancet. 2013;382:1993–2002. 10.1016/S0140-6736(13)61887-5 24055451 PMC3898949

